# Xp22.31 copy number variations in 87 fetuses: refined genotype–phenotype correlations by prenatal and postnatal follow-up

**DOI:** 10.1186/s12920-023-01493-z

**Published:** 2023-04-03

**Authors:** Huamei Hu, Yulin Huang, Renke Hou, Huanhuan Xu, Yalan Liu, Xueqian Liao, Juchun Xu, Lupin Jiang, Dan Wang

**Affiliations:** Department of Obstetrics and Gynecology, Southwest Hospital, Third Military Medical University (Army Medical University), Chongqing, China

**Keywords:** Xp22.31 deletion, Xp22.31 duplication, X-linked ichthyosis, Genetic counseling

## Abstract

**Background:**

Xp22.31 deletion and duplication have been described in various studies, but different laboratories interpret pathogenicity differently.

**Objectives:**

Our study aimed to refine the genotype–phenotype associations between Xp22.31 copy number variants in fetuses, with the aim of providing data support to genetic counseling.

**Methods:**

We retrospectively analyzed karyotyping and single nucleotide polymorphism array results from 87 fetuses and their family members. Phenotypic data were obtained through follow-up visits.

**Results:**

The percentage of fetuses carrying the Xp22.31 deletions (9 females, 12 males) was 24.1% (*n* = 21), while duplications (38 females, 28 males) accounted for 75.9% (*n* = 66). Here, we noted that the typical region (from 6.4 to 8.1 Mb, hg19) was detected in the highest ratio, either in the fetuses with deletions (76.2%, 16 of 21) or duplications (69.7%, 46 of 66). In female deletion carriers, termination of pregnancy was chosen for two fetuses, and the remaining seven were born without distinct phenotypic abnormalities. In male deletion carriers, termination of pregnancy was chosen for four fetuses, and the remaining eight of them displayed ichthyosis without neurodevelopmental anomalies. In two of these cases, the chromosomal imbalance was inherited from the maternal grandfathers, who also only had ichthyosis phenotypes. Among the 66 duplication carriers, two cases were lost at follow-up, and pregnancy was terminated for eight cases. There were no other clinical findings in the rest of the 56 fetuses, including two with Xp22.31 tetrasomy, for either male or female carriers.

**Conclusion:**

Our observations provide support for genetic counseling in male and female carriers of Xp22.31 copy number variants. Most of them are asymptomatic in male deletion carriers, except for skin findings. Our study is consistent with the view that the Xp22.31 duplication may be a benign variant in both sexes.

**Supplementary Information:**

The online version contains supplementary material available at 10.1186/s12920-023-01493-z.

## Introduction

Xp22.31 deletion is relatively common in the general population, with a carrier frequency of approximately 1 in 1500 males and 1 in 750 females [1, 2]. It was thought to be a pathogenic variant that contains the *STS* gene, the deletion of which causes X-linked ichthyosis (XLI). In 90% of XLI cases, it is caused by a deletion that completely encompasses the *STS* gene [3]. Clinical phenotypes include widespread dry, scaly skin and scaling. This condition affects males but is rarely reported in females [3]. Female deletion carriers have been reported to have a clinical phenotype of benign corneal opacities [4]. Recent studies have shown that the overall health and reproduction of heterozygous female carriers for Xp22.31 deletion exhibit apparently no or negligible differences compared to those of female non-carriers [2, 5]. Thus, the interpretation of female carriers is not controversial in prenatal diagnosis. For male carriers, all patients only had minor skin findings in some reports [6, 7]. In addition to ichthyosis, benign corneal opacities affected approximately 10–50% of males with XLI [8], and approximately 20% of males with XLI had cryptorchidism [2]. Furthermore, autism [9], intellectual disability [10–13], epilepsy [14], developmental delay [15], and kidney abnormalities [16] were described in male carriers of typical XLI-associated deletions (approximately 1.6 Mb). The Xp22.31 deletion is commonly classified as pathogenic according to the American College of Medical Genetics and Genomics recommendations for interpreting and reporting constitutional copy number variations (CNVs) [17], given its association with XLI. Despite the ascertained association, the skin phenotype can be improved with appropriate treatment and can be considered benign compared to other inherited dermatologic conditions or to the phenotypes associated with other pathogenic CNVs possibly detected in prenatal diagnosis, a setting in which the main attention is drawn on neurodevelopmental outcomes and structural anomalies. The possible association of Xp22.31 imbalances with neurodevelopmental phenotypes, reported by some authors [10–13] and excluded by others [6, 7], requires further studies. This makes the determination of pregnancy outcome difficult in case of male fetuses.

The frequency of Xp22.31 duplication has been reported to be as high as 0.41% in general population controls [18]. However, the pathogenicity of Xp22.31 duplication is debatable [19], some studies consider this duplication a variant of uncertain significance (VUS) [20, 21]. Recent studies interpret Xp22.31 duplication as benign [22, 23], whereas others suspect it is likely pathogenic. Some correlations include neurodevelopmental changes, intellectual disability, cognitive deficits, and seizures [24–28]. As a result, its interpretation varies between different diagnostic laboratories, which can lead to misdiagnosis. Our study aimed to provide more support for Xp22.31 genetic counseling by analyzing genotype–phenotype correlations in 87 cases.

## Materials and methods

### Subjects

This was a retrospective study at a tertiary referral center (Prenatal Diagnosis Center of Obstetrics and Gynecology, Southwest Hospital in Chongqing). Single nucleotide polymorphism (SNP) array testing and karyotyping were offered for fetal samples and post-birth information and childcare data were obtained via telephone follow-up assessment. By analyzing these data, 87 cases of Xp22.31 CNVs (21 deletions and 66 duplications) were selected from 13,568 cases of invasive prenatal diagnosis (amniocentesis and cordocentesis) between January 2016 and December 2021. Informed consent for invasive prenatal diagnosis was obtained from the parents before detection. This research was approved by the Ethics Committee of Southwest Hospital, Third Military Medical University (Army Medical University).

The ages of pregnant women at the time of prenatal diagnosis were between 20 and 38 years old. Amniocentesis was performed at a gestational age (GA) of 18–25 weeks. The pregnant women who chose cordocentesis had a GA between 28 and 32 weeks. Their indications included advanced maternal age, adverse pregnancy history, abnormalities of ultrasound, or non-invasive prenatal testing.

### Karyotyping

This procedure was described in our previous study [29].

### SNP array analysis

This procedure was described in our previous study [29]. The databases for analysis are as follows: DGV (http://dgv.tcag.ca/dgv/app/home), OMIM (http://www.ncbi.nlm.nih.gov/omim), gnomAD (http://gnomad-sg.org/), DECIPHER (https://www.deciphergenomics.org/), dbVar (http://www.ncbi.nlm.nih.gov/dbvar), ClinVar (http://www.ncbi.nlm.nih.gov/clinvar), ClinGen (https://www.ncbi.nlm.nih.gov/projects/dbvar/clingen/), and Pubmed. Benign or likely benign CNVs were not reported.

### Criteria for prenatal and postnatal follow-up assessment

#### Prenatal assessment

The results of ultrasound or MRI examination in the second and third trimesters of pregnancy were collected. Additionally, data on the frequency of pregnancies and births, pregnancy complications, and a history of adverse pregnancies were gathered.

#### Postnatal assessment

Data including mode of delivery, birth weight, length, combinations of neonatal diseases, and developmental details diagnosed by child healthcare professionals were collected. After obtaining the parents’ informed consent, the child healthcare data were collected to assess developmental details. General child healthcare was carried out by professional doctors in community hospitals, according to the World Health Organization’s physical and mental development table for infants aged 0–3 years.

Fetuses treated with terminated pregnancies: The cases and causes of termination of pregnancy (TOP), and the presence or absence of fetal anomalies were recorded.

## Results

### Genetic testing results

#### Cytogenetic results

Karyotype: All the 87 fetuses showed a normal karyotype.

#### SNP array results

Females with Xp22.31 deletions: Except for three cases with a deletion region smaller than 1 Mb, the segments of the remaining six cases were located in the typical 1.6 Mb deletion region (from 6.4 to 8.1 Mb, hg19), including *STS, PUDP, PNPLA4*, and *VCX* genes. A control analysis of parental SNP arrays was recommended and only one set of fetal parents performed this test, proving that the deletion was inherited maternally (Table 1).

**Table 1:** Summary female fetuses with Xp22.31 deletions

Number	Ultrasound findings	location of the CNV	Size	Protein-coding genes	Inheritance	Karyotye	Outcomes	birth with defects	Age at study (M)	Developmental disorders
1	Complex congenital heart disease	arr[GRCh37] Xp22.31(7819527_8432715) × 1	613 Kb	PNPLA4	NA	46, XX	TOP	–	–	–
2	/	arr[GRCh37] Xp22.31(6444607_8135053) × 1	1.69 Mb	PNPLA4, PUDP, STS, VCX, VCX3A	NA	46, XX	Born	/	53	/
3	/	arr[GRCh37] Xp22.31(6802248_7686400) × 1	884 Kb	PUDP, STS	NA	46,XX	Born	/	47	/
4	/	arr[GRCh37] Xp22.31(6643421_7157128) × 1	514 Kb	PUDP, STS	NA	46, XX	Born	/	44	/
5	Ventricular septal defect	arr[GRCh37] Xp22.31(6488784_8135053) × 1	1.65 Mb	PNPLA4, PUDP, STS, VCX	Inherited from mother	46, XX	Born	/	34	/
6	Cystic hygroma of the neck	arr[GRCh37] Xp22.31(6456940_8135053) × 1	1.68 Mb	PNPLA4, PUDP, STS, VCX	NA	46, XX	TOP	–	–	–
7	Echogenic intracardiac focus	arr[GRCh37] Xp22.31(6456940_8135053) × 1	1.68 Mb	PNPLA4, PUDP, STS, VCX	NA	46, XX	Born	/	8	/
8	/	arr[GRCh37] Xp22.31(6456940_8135053) × 1	1.68 Mb	PNPLA4, PUDP, STS, VCX	NA	46, XX	Born	/	6	/
9	/	arr[GRCh37] Xp22.31(6456940_8135053) × 1	1.68 Mb	PNPLA4, PUDP, STS, VCX	NA	46, XX	Born	/	3	/

*TOP* Termination of pregnancy

Males with Xp22.31 deletions: In male deletion carriers, the sizes of the loss regions were larger than 1 Mb in all fetuses (approximately 1.2 Mb in two cases and 1.6 Mb in 10 cases). Seven fetuses were identified as carrying the maternal genetic deletions (Table 2). In three of these, extended pedigree analysis showed that the maternal grandfathers carried the deletion in two cases, and, in the remaining case, the elder brother of the fetus was a carrier.

**Table 2:** Summary male fetuses with Xp22.31 deletions

Number	Ultrasound findings	location of the CNV	Size (Mb)	Protein-coding genes	Inheritance	Karyotype	Outcomes	birth with defects	Age at study (M)	Developmental disorders	Skin findings
1	Gastroschisis	arr[GRCh37] Xp22.31(6516735_8131442) × 0	1.61	PNPLA4, PUDP, STS, VCX	Inherited from the mother	46, XY	TOP	–	–	–	–
2	Cystic hygroma of the neck	arr[GRCh37] Xp22.31(6516735_8131442) × 0	1.61	PNPLA4, PUDP, STS, VCX	Inherited from the mother	46, XY	TOP	–	–	–	–
3	/	arr[GRCh37] Xp22.31(6456940_8131442) × 0	1.67	PNPLA4, PUDP, STS, VCX	Inherited from the mother	46, XY	TOP	–	–	–	–
4	/	arr[GRCh37] Xp22.31(6456940_8123407) × 0	1.67	PNPLA4, PUDP, STS, VCX	NA	46, XY	Born	/	54	/	dry, and polygonal scales on the abdomen, arms, and legs
5	/	arr[GRCh37] Xp22.31(6456940_8123407) × 0	1.67	PNPLA4, PUDP, STS, VCX	NA	46, XY	Born	/	50	/	dry, and polygonal scales on the abdomen, arms, and legs
6	/	arr[GRCh37] Xp22.31(6456940_8123407) × 0	1.67	PNPLA4, PUDP, STS, VCX	NA	46, XY	Born	/	50	/	dry, and polygonal scales
7	/	arr[GRCh37] Xp22.31(6456940_8123407) × 0	1.67	PNPLA4, PUDP, STS, VCX	Inherited from the mother	46, XY	TOP	–	–	–	–
8	Abnormal external genital development	arr[GRCh37] Xp22.31(6486490_123407) × 0	1.64	PNPLA4, PUDP, STS, VCX	Inherited from the mother and maternal grandfather	46, XY	Born	Hypospadias	44	/	mild ichthyosis
9	/	arr[GRCh37] Xp22.31(6486490_8123407) × 0	1.64	PNPLA4, PUDP, STS, VCX	Inherited from the mother, the elderbrother also carried	46, XY	Born	/	43	/	dry, and polygonal scales on the abdomen, arms, and legs
10	/	arr[GRCh37] Xp22.31(6631810_7837470) × 0	1.21	PUDP, STS, VCX	Inherited from the mother and maternal grandfather	46, XY	Born	/	33	/	mild ichthyosis
11	Double aortic arch	arr[GRCh37] Xp22.31(6681676_7874503) × 0	1.19	PNPLA4, PUDP, STS, VCX	NA	46, XY	Born	/	32	/	mild ichthyosis
12	/	arr[GRCh37] Xp22.31(6456940_8123407) × 0	1.67	PNPLA4, PUDP, STS, VCX	NA	46, XY	Born	/	29	/	ichthyosis symptoms are more serious

*NA* Not available, *TOP* Termination of pregnancy

Fetuses with Xp22.31 duplications: The typical 1.6 Mb duplicated segment occurred in 69.7% (46/66) of the fetuses. (sizes of the remaining 20 cases, one > 1.6 Mb and 19 < 1 Mb) (Additional files 1 and 2). Only 27.2% (18/66) of cases underwent parental SNP array testing, confirming that the duplications were inherited from their parents (Table 3). Most interestingly, two cases of Xp22.31 tetrasomy were identified in female fetuses. In one case, the parents were both heterozygous for a duplication, the fetus inherited two duplicated alleles, and thus four copies of the region. In the other case, the father had no copy number imbalances, while the mother and the fetus both carried a triplication of Xp22.31 and a normal allele.

**Table 3:** Summary fetuses with Xp22.31 duplications

	~ 1.9 Mb		~ 1.6 Mb						300 ~ 800 Kb		Total
	F	M	F			M			F	M	
TOP	/	/	2			3 (1^a^)			3	/	8
LF	/	/	1			/			/	1	2
Born	1	/	26			14			5	10	56
			11^a^	2^ab^	4^b^	3^a^	1^ab^	3^b^	2^b^	3^b^	
Total	1	/	29			17			8	11	66

*F* Female, *M* Male, *TOP* Termination of pregnancy, *LF* Loss at follow-up, ^a^inherited from the mother or father; ^b^the fetus with ultrasonographic soft markers

### Clinical follow-up outcomes

#### Xp22.31 deletions in females

TOP was chosen for two fetuses, one case was attributed to a severe ultrasonic finding. Seven females were born at full-term delivery, and two of them displayed ultrasonographic soft markers in pregnancy (ventricular septal defect, echogenic intracardiac focus) and were asymptomatic after birth. Of the seven female children, the youngest being 3-month old and the oldest 4-year and 5-month old at the time of the study, none received a diagnosis of developmental delay and intellectual disability from the children’s healthcare examinations, as reported by the families (Table 1).

#### Xp22.31 deletions in males

For four fetuses, the families opted for TOP. A total of eight fetuses were born. The youngest one was 2.5 years old and the oldest one was 4.5 years old at the time of the study. Except for ichthyosis, no other clinical consequences were found involving intelligence or neurodevelopment. Among the eight children with ichthyosis, one boy had severe symptoms and was treated in the dermatology department. For three of the male children, the main manifestation was mild ichthyosis without flaking, with remission in summer and a need for moisturizing care in winter. The skin disorders of the others were characterized by dry and polygonal scales on the abdomen, arms, and legs (Table 2). Similarly, other male family members who were confirmed to also carry Xp22.31 deletions did not have a phenotype of neurodevelopmental abnormalities, and only presented ichthyosis (Table 2). Abnormal external genital development was found in one male, but the maternal grandfather who carried the same deletion did not have this abnormality (Table 2).

#### Xp22.31 duplications in the fetuses

Among the 66 duplication carriers, two cases were lost at follow-up, and in eight fetuses, TOP was chosen. A total of 56 fetuses were born without any clinical defects, either male or female carriers. The youngest was 3 months old, and the oldest approximately 5.5 years old at the time of the study. In 15 of these cases with Xp22.31 duplication, ultrasonographic soft markers were detected during pregnancy. All of them were asymptomatic after birth (Table 3).

## Discussion

**Fig. 1:**
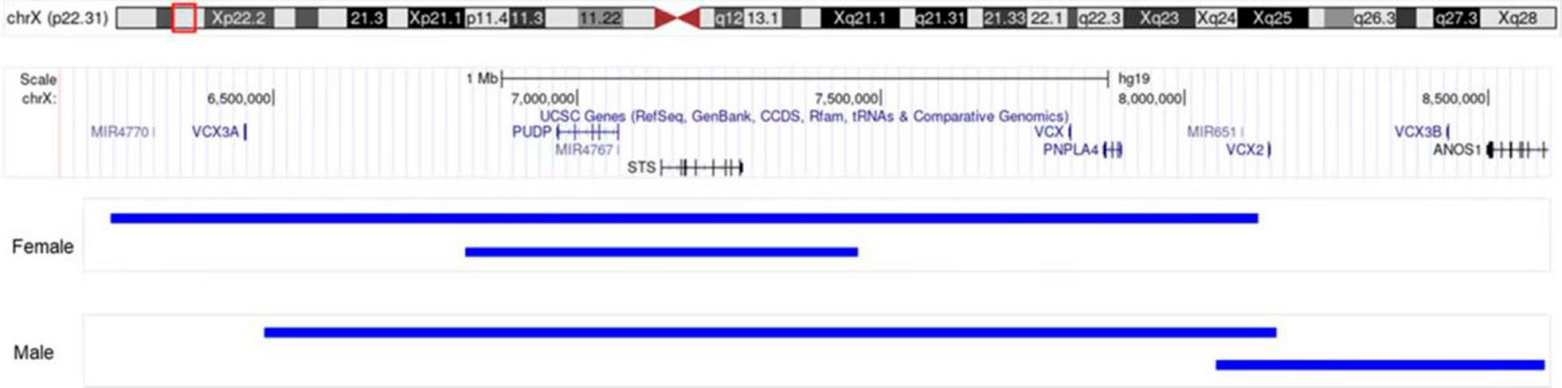
Genomic location of Xp22.31. The blue bars from top to bottom in orderrepresent the largest (arr[GRCh37] Xp22.31(6198422_8131442) × 3 1.93 Mb) and smallest duplicated segments of the female fetus in Xp22.31. (arr[GRCh37] Xp22.31(6901968_7379309) × 3 477 Kb); the largest segment (arr[GRCh37]Xp22.31(6456940_8135053) × 2 1.678 Mb) and the smallest segment of the male fetus (arr[GRCh37] Xp22.31(8253271_8590357) × 2 337 Kb)

The Xp22.31 segment of humans is a highly unstable region with frequent rearrangements [18]. Xp22.31 imbalances (including deletions and duplications) are among the most frequently detected CNVs in prenatal diagnosis. The typical deletion at Xp22.31 is approximately 1.6 Mb in size and encompasses the *STS, HDHD1/ PUDP, PNPLA4*, and *VCX* protein-encoding genes (Fig. 1). It is classified as pathogenic according to the American College of Medical Genetics and Genomics recommendations [17] and leads to ichthyosis, which mainly affects males. Therefore, genetic counseling differs for males and females. However, this difference between sexes does not exist in the corresponding Xp22.31 duplication, the pathogenicity of which is still debatable.

In our findings, all female deletion carriers had a normal phenotype after birth. Although corneal opacities are reported to affect approximately 10–50% of males with XLI and approximately 25% of female carriers [2], this phenotype was absent both in the males and females in our study. This may be because the detection of corneal opacities is quite rare before puberty [30]. As the *HDHD1A*, *PNPLA4*, and *STS* genes have been shown to escape X-inactivation [31], females with Xp22.31 deletion contain the same number of active *STS* alleles as healthy males, in whom skin scales are rare [30, 32]. This seems to explain the asymptomatic nature of females with Xp22.31 deletion in our study. Large cohort studies have demonstrated that the phenotypic differences between female Xp22.31 deletion carriers and non-carriers are negligible [2, 5]. Thus, the interpretation of female carriers is not controversial in prenatal diagnosis.

The content of adjacent genes is closely related to the phenotypes. The extensive deletions encompassing more nearby genes are associated with severe conditions, the phenotypes of which include ocular albinism, epilepsy, abnormal electroencephalography, intellectual disability, hyposmia, attention deficit hyperactivity disorder, autism, and language development disorder [9, 33, 34]. The typical deletion (approximately 1.6 Mb) encompasses *STS* and a small number of adjacent genes (*PUDP*, *PNPLA4*, and *VCX*)*, STS* is labeled as a haploinsufficient gene (ClinGen haploinsufficiency score 3, sufficient evidence supporting haploinsufficiency. last accessed: January 20th, 2023), which is responsible for XLI. It has been described to be highly expressed in the adult brain and may have implications for neurodevelopment and ongoing brain function via a number of direct and indirect mechanisms [35]. Adult STS-deficient male mice exhibit substantially altered striatal neurochemistry [36]. Previous studies have suggested that *STS* deficiency plays a direct role in the pathogenesis of attention deficit hyperactivity disorder [30]. Notably, in public databases, patients with deletions only containing the *STS* gene also showed the phenotype of intellectual disability (Decipher:283,235, 350,438). The CNVs in one case was classified as pathogenic (350,438), while in another was not recorded (283,235). Chatterjee et al. suggested that individuals lacking *STS* are at increased risk of developmental and mood disorders [35]. No haploinsufficiency sensitivity score was available on ClinGen for *PUDP*, *VCX* and *PNPLA4* (last accessed: January 20th, 2023). The *VCX* proteins affect proper neuritogenesis [37]. Studies have shown that the absence of the *VCX* gene could contribute to an intellectual disability phenotype [37]. Labonne et al. proposed that *HDHD1*/*PUDP* and *PNPLA4* play a role in X-linked intellectual disability [13], because of their high transcript levels in the human brain [13].

Previous studies have reported the loss of this region with or without neurodevelopmental abnormalities [7, 12]. Moreover, in our observations, the deletion of the *HDHD1A*, *PNPLA4*, *VCX*, and *STS* genes was not associated with mental development traits. Cryptorchidism was not found in male fetuses, however, abnormal external genital development was described in one of them, but the maternal grandfather who carried the same deletion did not exhibit this abnormality. Some pregnant women in our study chose TOP because of the risk of mental defects in male fetuses after birth, but not skin disorders. Reduced penetrance and expression variability may contribute to phenotype variability, and even the correlation between the deletion and neurodevelopmental abnormalities requires further confirmation.

Although Xp22.31duplication has been described in various studies, the classification of pathogenicity remains inconsistent. It has been interpreted in some cases as a VUS [20, 21] or benign [22, 23], and in others as a cause of developmental disorders, including autism, intellectual disability, cognitive deficits, and seizures [24–28], these phenotypic traits were identified in both males and females with no significant gender differences. With the exception of *STS* (ClinGen triplosensitivity score 0, no evidence supporting triplosensitivity), *PUDP*, *VCX*, and *PNPLA4* did not have an entry on ClinGen. (last accessed: January 20th, 2023). Many duplication carriers with neurodevelopmental phenotypes appeared in the DECIPHER database. The individuals carrying smaller duplicated segments, which are around 100 kb and contain the *STS* gene only, have a phenotype of intellectual disability (359,225, 256,781). However, the pathogenicity of this CNV is still unclear.

In our study, the follow-up of 56 fetuses with Xp22.31 duplications after birth showed no developmental disorders, epilepsy, and other symptoms. In two male fetuses, the imbalances were inherited from the mothers. Polo-Antúnez et al. described a severe neurological phenotype in a girl with Xp22.31 tetrasomy [38]. In contrast, we identified no abnormal findings in two females and a mother, all of whom had Xp22.31 tetrasomy. Whether the absence of disease phenotypic features in our cases is related to other modifying factors in the genomic background, such as reduced penetrance and efficiency of genes escaping X-inactivation is unclear. However, our observations are consistent with the view that Xp22.31 duplication (from 6.4 to 8.1 Mb, hg19), including the *STS*, *PUDP*, *PNPLA4*, and *VCX* genes, is likely to be a benign CNV.

Ultrasonographic soft markers during pregnancy, such as ventricular echoic spot and single umbilical artery were detected in our study, and seemed to have no correlation with Xp22.31deletion and duplication. First-trimester fetal cystic hygroma was the indication for invasive prenatal testing in some of the cases later detected with Xp22.31 imbalances. Cystic hygroma presents a high risk of aneuploidies [39]. Euploid fetuses with cystic hygroma also present an increased risk for submicroscopic CNVs [40] and specific monogenic disorders such as Rasopathies [41]. At present, the association of cystic hygroma with Xp22.31 is unknown. Genome/exome sequencing was recommended for fetuses with complex congenital heart disease or gastroschisis.

In conclusion, our study provided more benign evidence for the approximately 1.6 Mb typical Xp22.31 duplications and Xp22.31 deletions in female. Although Xp22.31 deletion is generally assessed as pathogenic in many genetic laboratories, genetic counseling for male and female fetuses should be differentiated. The deletion in females is likely a benign variant. Genetic counseling for male fetuses is challenging. The skin disorders can be improved with appropriate treatment. In the current state of knowledge, the Xp22.31 deletion can also be considered in males as a susceptibility factor for neurodevelopmental disorders. The possible association of Xp22.31 imbalances with neurodevelopmental phenotypes, reported by some authors and excluded by others, requires further studies. However, a professional explanation of the risk of neurodevelopmental abnormalities is key to avoid causing anxiety in pregnant women. We advocate multi-disciplinary care after birth, including neurology, pediatrics, and dermatology.

## Supplementary Information

**Additional file1 Table S1.** Characterization of the Xp22.31 duplication for each female fetus

**Additional file 2 Table S2. **Characterization of the Xp22.31 duplication for each male fetus

## Data Availability

The public database for supporting the findings of this study as follows: DGV (http://dgv.tcag.ca/dgv/app/home), OMIM (http://www.ncbi.nlm.nih.gov/omim), gnomAD (http://gnomad-sg.org/), DECIPHER (https://www.deciphergenomics.org/), dbVar (http://www.ncbi.nlm.nih.gov/dbvar), ClinVar (http://www.ncbi.nlm.nih.gov/clinvar), ClinGen (https://www.ncbi.nlm.nih.gov/projects/dbvar/clingen/), and Pubmed. To view each patient with details visit https: http://decipher.sanger.ac.uk (283,235, 350,438, 359,225,256,781).
