# Supplementary material for: Xp22.31 copy number variations in 87 fetuses: refined genotype–phenotype correlations by prenatal and postnatal follow-up
Source: BMC Med Genomics. 2023 Apr 3;16:69. doi: 10.1186/s12920-023-01493-z (PMC10069036; doi:10.1186/s12920-023-01493-z)
Supplement: Supplementary file 1 — Additional file1 Table S1. Characterization of the Xp22.31 duplication for each female fetus [file 12920_2023_1493_MOESM1_ESM.docx]

| Supplementary Table 1. Characterization of the Xp22.31 duplication for each  female fetus | | | |
| --- | --- | --- | --- |
| Fetus | Location of the CNV | Size | Protein-coding genes |
| 1 | arr[GRCh37] Xp22.31(6198422_8131442)×3 | 1.93Mb | PNPLA4, PUDP, STS, VCX VCX3A |
| 2 | arr[GRCh37] Xp22.31(6755393_7537276)×3 | 782Kb | PUDP, STS |
| 3 | arr[GRCh37] Xp22.31(6444607_8135053)×3 | 1.69Mb | PNPLA4, PUDP, STS, VCX VCX3A |
| 4 | arr[GRCh37] Xp22.31(6444607_8126894)×3 | 1.68Mb | PNPLA4, PUDP, STS, VCX VCX3A |
| 5 | arr[GRCh37] Xp22.31(6418059_8126894)×3 | 1.71Mb | PNPLA4, PUDP, STS, VCX VCX3A |
| 6 | arr[GRCh37] Xp22.31(6456940_8135053)×4 | 1.68Mb | PNPLA4, PUDP, STS, VCX |
| 7 | arr[GRCh37] Xp22.31(6456940_8163965)×3 | 1.71Mb | PNPLA4, PUDP, STS, VCX |
| 8 | arr[GRCh37] Xp22.31(6486490_8135053)×3 | 1.65Mb | PNPLA4, PUDP, STS, VCX |
| 9 | arr[GRCh37] Xp22.31(6488784_8135053)×3 | 1.65Mb | PNPLA4, PUDP, STS, VCX |
| 10 | arr[GRCh37] Xp22.31(6456940_8135053)×3 | 1.68Mb | PNPLA4, PUDP, STS, VCX |
| 11 | arr[GRCh37] Xp22.31(6456940_8135053)×3 | 1.68Mb | PNPLA4, PUDP, STS, VCX |
| 12 | arr[GRCh37] Xp22.31(7323930_8135053)×3 | 811Kb | PUDP, STS, VCX |
| 13 | arr[GRCh37] Xp22.31(6755393_7422760)×3 | 667Kb | PUDP, STS |
| 14 | arr[GRCh37] Xp22.31(6456940_8135053)×3 | 1.68Mb | PNPLA4, PUDP, STS, VCX |
| 15 | arr[GRCh37] Xp22.31(6488784_7177443)×3 | 689Kb | STS |
| 16 | arr[GRCh37] Xp22.31(6488784_7177443)×3 | 689Kb | STS |
| 17 | arr[GRCh37] Xp22.31(6513884_8135053)×3 | 1.62Mb | PNPLA4, PUDP, STS, VCX |
| 18 | arr[GRCh37] Xp22.31(6456940_8135053)×3 | 1.68Mb | PNPLA4, PUDP, STS, VCX |
| 19 | arr[GRCh37] Xp22.31(6488784_8165655)×3 | 1.68Mb | PNPLA4, PUDP, STS, VCX |
| 20 | arr[GRCh37] Xp22.31(6513884_8135053)×3 | 1.62Mb | PNPLA4, PUDP, STS, VCX |
| 21 | arr[GRCh37] Xp22.31(6513884_8135053)×3 | 1.62Mb | PNPLA4, PUDP, STS, VCX |
| 22 | arr[GRCh37] Xp22.31(6901968_7379309)×3 | 477Kb | STS |
| 23 | arr[GRCh37] Xp22.31(6513884_8135053)×3 | 1.62Mb | PNPLA4, PUDP, STS, VCX |
| 24 | arr[GRCh37] Xp22.31(6513884_8135053)×3 | 1.62Mb | PNPLA4, PUDP, STS, VCX |
| 25 | arr[GRCh37] Xp22.31(6500342_8168701)×3 | 1.68Mb | PNPLA4, PUDP, STS, VCX,VCX2 |
| 26 | arr[GRCh37] Xp22.31(6490626_8135053)×3 | 1.64Mb | PNPLA4, PUDP, STS, VCX |
| 27 | arr[GRCh37] Xp22.31(6490626_8135053)×4 | 1.64Mb | PNPLA4, PUDP, STS, VCX |
| 28 | arr[GRCh37] Xp22.31(6488784_8135053)×3 | 1.65Mb | PNPLA4, PUDP, STS, VCX |
| 29 | arr[GRCh37] Xp22.31(6490626_8135053)×3 | 1.64Mb | PNPLA4, PUDP, STS, VCX |
| 30 | arr[GRCh37] Xp22.31(6490626_8135053)×3 | 1.64Mb | PNPLA4, PUDP, STS, VCX |
| 31 | arr[GRCh37] Xp22.31(6490626_8135053)×3 | 1.64Mb | PNPLA4, PUDP, STS, VCX |
| 32 | arr[GRCh37] Xp22.31(6490626_8135053)×3 | 1.64Mb | PNPLA4, PUDP, STS, VCX |
| 33 | arr[GRCh37] Xp22.31(6490626_8135053)×3 | 1.64Mb | PNPLA4, PUDP, STS, VCX |
| 34 | arr[GRCh37] Xp22.31(6456940_8135053)×3 | 1.68Mb | PNPLA4, PUDP, STS, VCX |
| 35 | arr[GRCh37] Xp22.31(6980443_7752936)×3 | 772Kb | STS |
| 36 | arr[GRCh37] Xp22.31(6456940_7204036)×3 | 747kb | PUDP, STS |
| 37 | arr[GRCh37] Xp22.31(6418059_8135053)×3 | 1.72Mb | PNPLA4, PUDP, STS, VCX VCX3A |
| 38 | arr[GRCh37] Xp22.31(6456940_8135053)×3 | 1.68Mb | PNPLA4, PUDP, STS, VCX |
