# Supplementary material for: Xp22.31 copy number variations in 87 fetuses: refined genotype–phenotype correlations by prenatal and postnatal follow-up
Source: BMC Med Genomics. 2023 Apr 3;16:69. doi: 10.1186/s12920-023-01493-z (PMC10069036; doi:10.1186/s12920-023-01493-z)
Supplement: Supplementary file 2 — Additional file 2 Table S2. Characterization of the Xp22.31 duplication for each male fetus [file 12920_2023_1493_MOESM2_ESM.docx]

| Supplementary Table 2. Characterization of the Xp22.31 duplication for each male fetus | | | |
| --- | --- | --- | --- |
| Fetus | Location of the CNV | Size | Protein-coding genes |
| 1 | Xp22.31(6444607_8135053)×2 | 1.70Mb | PNPLA4, PUDP, STS, VCX VCX3A |
| 2 | Xp22.31(6486490_8135053)×2 | 1.65Mb | PNPLA4, PUDP, STS, VCX |
| 3 | Xp22.31(6486490_8135053)×2 | 1.65Mb | PNPLA4, PUDP, STS, VCX |
| 4 | Xp22.31(6486490_8135053)×2 | 1.65Mb | PNPLA4, PUDP, STS, VCX |
| 5 | Xp22.31(7023678_7745631)×2 | 722Kb | STS |
| 6 | Xp22.31(6054885_6500394)×2 | 446Kb | NLGN4X,VCX3A |
| 7 | Xp22.31(6486490_8135053)×2 | 1.65Mb | PNPLA4, PUDP, STS, VCX |
| 8 | Xp22.31(6486490_8135053)×2 | 1.65Mb | PNPLA4, PUDP, STS, VCX |
| 9 | Xp22.31(6486490_8135053)×2 | 1.65Mb | PNPLA4, PUDP, STS, VCX |
| 10 | Xp22.31(6486490_8135053)×2 | 1.65Mb | PNPLA4, PUDP, STS, VCX |
| 11 | Xp22.31(6490626_8135053)×2 | 1.64Mb | PNPLA4, PUDP, STS, VCX |
| 12 | Xp22.31(6490626_8135053)×2 | 1.64Mb | PNPLA4, PUDP, STS, VCX |
| 13 | Xp22.31(6807437_7157128)×2 | 350Kb | PUDP, STS |
| 14 | Xp22.31(6490626_8168701)×2 | 1.68Mb | PNPLA4, PUDP, STS, VCX,VCX2 |
| 15 | Xp22.31(6490626_8135053)×2 | 1.64Mb | PNPLA4, PUDP, STS, VCX |
| 16 | Xp22.31(6490626_8135053)×2 | 1.64Mb | PNPLA4, PUDP, STS, VCX |
| 17 | Xp22.31(6975782_7743257)×2 | 767Kb | STS |
| 18 | Xp22.31(6456940_8135053)×2 | 1.68Mb | PNPLA4, PUDP, STS, VCX |
| 19 | Xp22.31(6456940_7379309)×2 | 922Kb | PUDP, STS |
| 20 | Xp22.31(8253271_8590357)×2 | 337Kb | VCX3B,ANOS1 |
| 21 | Xp22.31(8253271_8590357)×2 | 337Kb | VCX3B,ANOS1 |
| 22 | Xp22.31(8253271_8590357)×2 | 337Kb | VCX3B,ANOS1 |
| 23 | Xp22.31(6456940_8135053)×2 | 1.68Mb | PNPLA4, PUDP, STS, VCX |
| 24 | Xp22.31(7850717_8394253)×2 | 544kb | PNPLA4 |
| 25 | Xp22.31(6456940_7223150)×2 | 766kb | VCX3A,PUDP, STS |
| 26 | Xp22.31(6456940_8135053)×2 | 1.68Mb | PNPLA4, PUDP, STS, VCX |
| 27 | ,Xp22.31(6456940_8135053)×2 | 1.68Mb | PNPLA4, PUDP, STS, VCX |
| 28 | Xp22.31(6456940_7225221)×2 | 768kb | VCX3A,PUDP, STS |
